# The lactate gap as a diagnostic clue in ethylene glycol poisoning and a tool for differentiating methanol intoxication

**DOI:** 10.1590/2175-8239-JBN-2026-0062en

**Published:** 2026-10-05

**Authors:** Koody André Hassemi Kitawara, Carlos Alberto Balda, Érika Bevilaqua Rangel

**Affiliations:** 1 Universidade Federal de São Paulo, Departamento de Medicina, Disciplina de Nefrologia, São Paulo, SP, Brazil.

Dear Editor,

We read with great interest the review by Nogueira et al. regarding the potential mechanisms of acute kidney injury following methanol intoxication, including oxidative stress, cell death, tissue hypoxia, and elevated lactate levels^1^. Poisoning by toxic alcohols, such as methanol, ethylene glycol (EG), isopropanol, diethylene glycol, and propylene glycol, is potentially fatal and may result in both renal and neurological impairment^2^.

Accidental or intentional ingestion of these substances may also lead to severe high anion gap metabolic acidosis, primarily driven by the accumulation of toxic metabolites, particularly glycolic acid in EG poisoning and formic acid in methanol poisoning^2,3^. Early diagnosis is therefore critical, although often challenging when direct measurement of toxic alcohols or their metabolites are unavailable.

In this context, the lactate gap, defined as a discrepancy between markedly elevated point-of-care (POC) lactate concentrations and normal or minimally increased laboratory lactate values, represents a valuable diagnostic clue, particularly in EG intoxication^3^. When present alongside urinary calcium oxalate crystals, it can strongly support the diagnosis and facilitate early recognition and treatment. Importantly, the lactate gap may also help differentiate EG poisoning from methanol intoxication, in which elevated lactate levels usually reflect true lactic acidosis secondary to tissue hypoxia and mitochondrial dysfunction rather than analytical interference.

In emergency settings, POC blood gas analyzers commonly measure lactate using the lactate oxidase (LOD) method, which detects hydrogen peroxide (H_2_O_2_) generated during lactate oxidation. In contrast, central laboratory analyzers typically employ the lactate dehydrogenase (LDH) method, which quantifies reduced nicotinamide adenine dinucleotide (NADH) production^4,5^.

From a biochemical standpoint, L-lactate measurement depends on its conversion to pyruvate, a reaction catalyzed either by LOD, producing H_2_O_2_, or by LDH, generating NADH^5^. Falsely elevated L-lactate concentrations are observed with LOD-based assays but not with LDH-based methods, indicating that the analytical interference is specific to LOD.

Although glycolic acid lacks the methyl group present in L-lactate (Figure 1), its core carbon structure is sufficiently similar to interact with LOD. This interaction leads to H_2_O_2_ generation and, consequently, to spuriously elevated lactate readings on POC analyzers.

**Figure 1:**
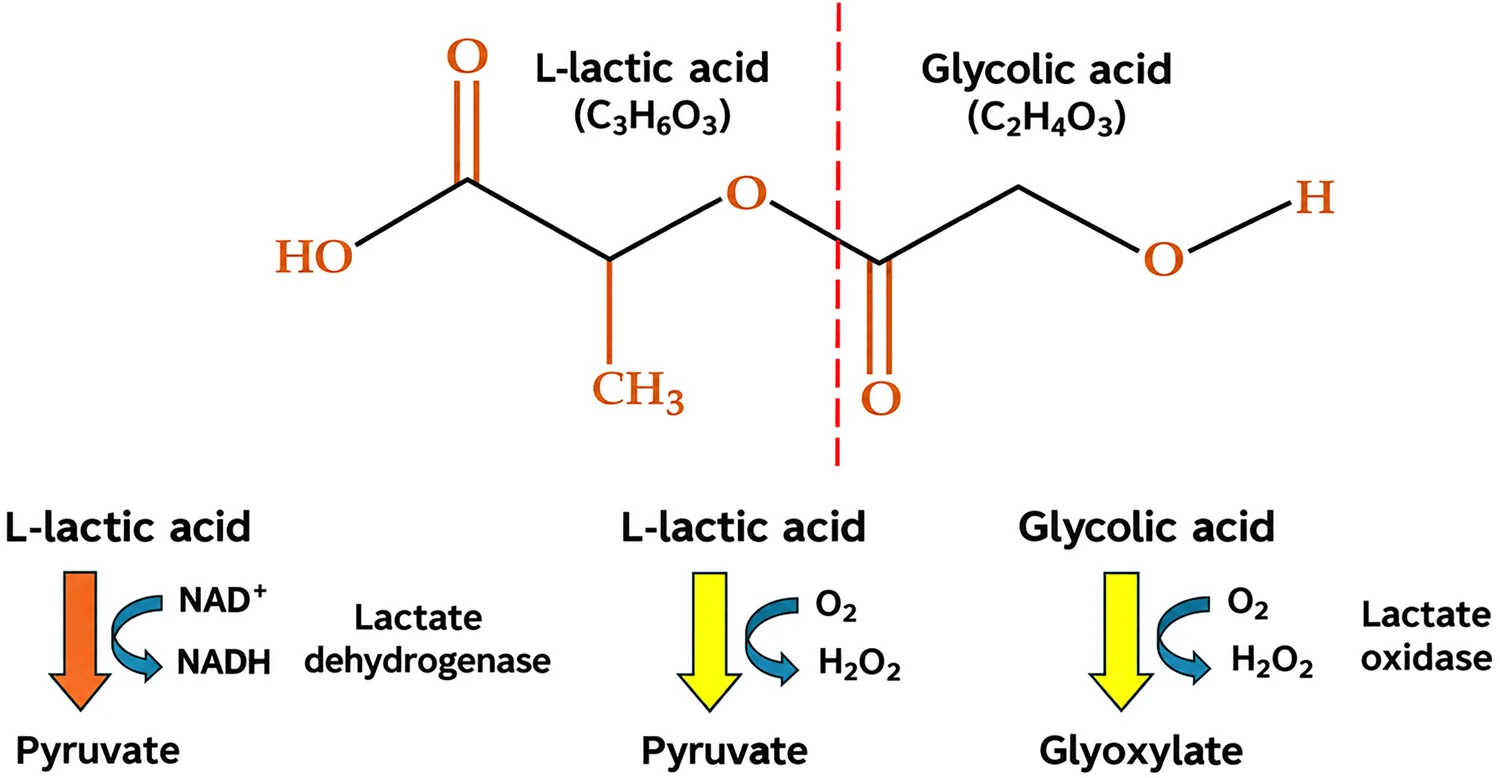
Structural difference between L-lactic acid (C_3_H_6_O_3_) and glycolic acid (C_2_H_4_O_3_). L-lactic acid has three carbons and includes a methyl group (–CH_3_), whereas glycolic acid has two carbons and does not have a methyl group. Because glycolic acid lacks a methyl group, its carbon backbone closely resembles that of L-lactate. This structural similarity allows glycolic acid to cross-react with lactate oxidase (LOD), generating hydrogen peroxide (H_2_O_2_) and falsely elevating lactate readings on point-of-care (POC) analyzers, an interference not observed with lactate dehydrogenase (LDH)–based assays.

This analytical artifact has important clinical implications. In patients presenting with high anion gap metabolic acidosis, a falsely elevated POC lactate may lead clinicians to attribute the acidosis to sepsis or hypoperfusion, thereby delaying consideration of toxic alcohol ingestion and postponing initiation of appropriate antidotal therapy. Recognition of a lactate gap, particularly when accompanied by an evolving osmolar gap, should prompt urgent evaluation for EG poisoning and early initiation of alcohol dehydrogenase inhibition, with fomepizole or ethanol, in conjunction with high-flux hemodialysis, even before confirmatory testing is available. In addition, the lactate gap may serve as a useful adjunct for therapeutic monitoring, as treatment can be discontinued once the anion gap normalizes (<18 mmol/L), acidosis resolves, serum EG and methanol concentrations fall below 25 and 20 mg/dL, respectively, and the lactate gap decreases to <2 mmol/L^2,6^.

In conclusion, early recognition of the triad of high anion gap metabolic acidosis, osmolar gap, and lactate gap is critical for survival in EG intoxication and helps distinguish it from methanol poisoning, in which lactate elevation generally reflects true metabolic derangement. The lactate gap may serve as a practical surrogate marker for both diagnosis and therapeutic guidance, as prompt initiation of antidotal therapy and hemodialysis substantially reduces morbidity and mortality.

## References

[1] Nogueira GN, Teles CBM, Varao MVS, Costa AC, Ramos GML, Daher EF (2026). Methanol intoxication and acute kidney injury: pathophysiological
mechanisms and therapeutic approaches. J Bras Nefrol.

[2] Kraut JA, Mullins ME (2018). Toxic alcohols. N Engl J Med.

[3] Kitawara KAH, Cerqueira BP, Balda CA, Rangel EB (2026). Severe ethylene glycol intoxication with marked lactate and
osmolar gaps leading to acute kidney injury: a case report. Int J Emerg Med.

[4] Brindley PG, Butler MS, Cembrowski G, Brindley DN (2007). Falsely elevated point-of-care lactate measurement after
ingestion of ethylene glycol. CMAJ.

[5] Sagar AS, Jimenez CA, McKelvy BJ (2018). Lactate gap as a tool in identifying ethylene glycol
poisoning. BMJ Case Rep.

[6] Simhadri PK, Daggula NR, Murari U, Singh P, Pantangi V, Chandramohan D (2024). Lactate gap: a clinical tool for diagnosing and managing ethylene
glycol poisoning. JEM Rep.

