# Supplementary material for: The lactate gap as a diagnostic clue in ethylene glycol poisoning and a tool for differentiating methanol intoxication
Source: J Bras Nefrol. 2026 Oct 5;48(4):e20260062. doi: 10.1590/2175-8239-JBN-2026-0062en (PMC13638184; doi:10.1590/2175-8239-JBN-2026-0062en)
Supplement: Translation

# Translation: A discrepância nas dosagens de lactato como pista diagnóstica na intoxicação por etilenoglicol e ferramenta para diferenciar a intoxicação por metanol

**DOI:** 10.1590/2175-8239-JBN-2026-0062pt

**Authors:** Koody André Hassemi Kitawara, Carlos Alberto Balda, Érika Bevilaqua Rangel

**Affiliations:** 1 Universidade Federal de São Paulo, Departamento de Medicina, Disciplina de Nefrologia, São Paulo, SP, Brasil.

Prezado Editor,

Lemos com grande interesse a revisão de Nogueira et al. sobre os potenciais mecanismos da lesão renal aguda após intoxicação por metanol, incluindo estresse oxidativo, morte celular, hipóxia tecidual e elevação dos níveis de lactato^1^. A intoxicação por álcoois tóxicos, como metanol, etilenoglicol (EG), isopropanol, dietilenoglicol e propilenoglicol, é potencialmente fatal e pode resultar em comprometimento renal e neurológico^2^.

A ingestão acidental ou intencional dessas substâncias também pode causar acidose metabólica grave com aumento do ânion gap, decorrente principalmente do acúmulo de metabólitos tóxicos, especialmente o ácido glicólico na intoxicação por EG e o ácido fórmico na intoxicação por metanol^2,3^. O diagnóstico precoce é, portanto, fundamental, embora frequentemente desafiador quando a mensuração direta dos álcoois tóxicos ou seus metabólitos não está disponível.

Nesse contexto, a discrepância nas dosagens de lactato (*lactate gap*), definida como a discordância entre concentrações marcadamente elevadas de lactato obtidas por analisadores *point-of-care* (POC) e valores normais ou discretamente elevados medidos por métodos laboratoriais convencionais, representa uma importante pista diagnóstica, particularmente na intoxicação por EG^3^. Quando presente em associação com cristais urinários de oxalato de cálcio, pode reforçar significativamente o diagnóstico e favorecer o reconhecimento e o tratamento precoces. Além disso, essa discrepância nas dosagens de lactato pode auxiliar na diferenciação entre intoxicação por EG e intoxicação por metanol, na qual a elevação do lactato geralmente reflete uma acidose láctica verdadeira secundária à hipóxia tecidual e à disfunção mitocondrial, e não uma interferência analítica.

Nos serviços de emergência, os analisadores de gasometria utilizados à beira do leito (POC) geralmente medem o lactato por meio do método da lactato oxidase (LOD), que detecta o peróxido de hidrogênio (H_2_O_2_) gerado durante a oxidação do lactato. Em contraste, os analisadores de laboratórios centrais empregam, em sua maioria, o método da lactato desidrogenase (LDH), que quantifica a produção de nicotinamida adenina dinucleotídeo reduzida (NADH)^4,5^.

Do ponto de vista bioquímico, a mensuração do L-lactato depende de sua conversão em piruvato, reação catalisada pela LOD, com produção de H_2_O_2_, ou pela LDH, com geração de NADH^5^. Concentrações falsamente elevadas de L-lactato são observadas em ensaios baseados na LOD, mas não nos métodos baseados na LDH, indicando que a interferência analítica é específica da LOD.

Embora o ácido glicólico não possua o grupo metila presente no L-lactato (Figura 1), sua estrutura carbônica central é suficientemente semelhante para interagir com a LOD. Essa interação leva à produção de H_2_O_2_ e, consequentemente, à elevação espúria das concentrações de lactato nos analisadores POC.

**Figura 1:** Diferença estrutural entre o ácido L-láctico (C_3_H_6_O_3_) e o ácido glicólico (C_2_H_4_O_3_). O ácido L-láctico possui três átomos de carbono e contém um grupo metila (–CH_3_), enquanto o ácido glicólico possui dois átomos de carbono e não apresenta grupo metila. Devido à ausência desse grupo, a cadeia carbônica do ácido glicólico apresenta grande semelhança estrutural com a do ácido L-láctico. Essa similaridade permite que o ácido glicólico apresente reação cruzada com a lactato oxidase (LOD), gerando peróxido de hidrogênio (H_2_O_2_) e causando elevação falsa das concentrações de lactato em analisadores *point-of-care* (POC), interferência que não ocorre em ensaios baseados na lactato desidrogenase (LDH).

Esse artefato analítico possui importantes implicações clínicas. Em pacientes com acidose metabólica com aumento do ânion gap, um valor falsamente elevado de lactato obtido por POC pode levar o clínico a atribuir a acidose à sepse ou à hipoperfusão, retardando a suspeita de intoxicação por álcoois tóxicos e, consequentemente, o início da terapia antidótica apropriada. O reconhecimento da discrepância nas dosagens de lactato, especialmente quando acompanhada de aumento progressivo do gap osmolar, deve motivar investigação imediata para intoxicação por EG e o início precoce da inibição da álcool desidrogenase, com fomepizol ou etanol, em associação à hemodiálise de alto fluxo, mesmo antes da confirmação laboratorial. Além disso, a discrepância nas dosagens de lactato pode constituir um marcador auxiliar útil para o monitoramento terapêutico, uma vez que o tratamento pode ser suspenso quando o ânion gap se normalizar (<18 mmol/L), a acidose for corrigida, as concentrações séricas de EG e metanol forem inferiores a 25 e 20 mg/dL, respectivamente, e a discrepância nas dosagens de lactato diminuir para <2 mmol/L^2,6^.

Em conclusão, o reconhecimento precoce da tríade composta por acidose metabólica com aumento do ânion gap, gap osmolar e discrepância nas dosagens de lactato é essencial para a sobrevida de pacientes com intoxicação por EG e pode auxiliar na diferenciação da intoxicação por metanol, na qual a elevação do lactato geralmente reflete um distúrbio metabólico verdadeiro. A discrepância nas dosagens de lactato pode representar um marcador substituto prático tanto para o diagnóstico quanto para a orientação terapêutica, uma vez que o início imediato da terapia antidótica e da hemodiálise reduz substancialmente a morbidade e a mortalidade.
